# Ergebnisse einer Umfrage zur Zusatzweiterbildung Ernährungsmedizin – Handlungsbedarf erkennbar

**DOI:** 10.1055/a-2737-2113

**Published:** 2026-01-12

**Authors:** Elisabeth Blüthner, Jamal Ali

**Affiliations:** 1 14903 Medizinische Klinik m.S. Hepatologie und Gastroenterologie CCM/CVK, Charité - Universitätsmedizin Berlin, Berlin, Germany; 2 522475 Berlin Institute of Health at Charité, Berlin, Germany; 3 Kliniken Rhein-Berg, Marien-Krankenhaus GFO, Gladbach, Germany

**Keywords:** Zusatzweiterbildung, Ernährungsmedizin, Umfrage, additional qualification, nutritional medicine, survey

## Abstract

**Hintergrund:**

Ernährungsmedizin ist ein wichtiger Bereich der Gastroenterologie. Dafür ist geschultes ärztliches Personal erforderlich, welches sich durch die Zusatzweiterbildung Ernährungsmedizin qualifiziert.

**Methodik:**

Im Herbst 2023 wurde eine Umfrage unter jungen Gastroenterolog*innen (JUGA) der DGVS durchgeführt. Es wurden demografische Daten, einrichtungsbezogener und persönlicher Stellenwert der Ernährungsmedizin und Zusatzweiterbildung mittels Likert-Skalen (Bewertungsskala 1 bis 10), Single- und Multiple-Choice-Fragen abgefragt. Zudem wurde der Einfluss von Geschlecht, Alter, Position und Einrichtung untersucht.

**Ergebnisse:**

Der persönliche Stellenwert der Ernährungsmedizin der 225 Teilnehmer*innen war im Median 8 (7–10), der Stellenwert in der tätigen Einrichtung 5 (3–7). Dieser wurde in Universitätskliniken signifikant (p=0,012) höher bewertet als an nicht-universitären Einrichtungen. Als Hürden werden ein unklarer Ablauf der Zusatzweiterbildung, Verfügbarkeit der Fallseminare und Kurse sowie fehlende Unterstützung auf Führungsebene angegeben.

**Schlussfolgerung:**

Der Stellenwert der Ernährungsmedizin ist an klinischen Einrichtungen niedriger als das persönliche Interesse an der Ernährungsmedizin und Zusatzweiterbildung, diese ist jedoch aktuell mit signifikanten Hürden verbunden. Strukturelle Verbesserungen und stärkere Anerkennung sind erforderlich, um die Zusatzweiterbildung weiterhin attraktiv zu machen und die größer werdende Nachfragenlücke zu decken.

## Abstract (en)

**Background:**

Nutritional medicine is an important area of gastroenterology. It requires trained medical personnel who have qualified through additional training in nutritional medicine.

**Methodology:**

In fall 2023, a survey was conducted among young gastroenterologists (JUGA) of the DGVS. Demographic data, the institutional and personal importance of nutritional medicine, and additional training were surveyed using Likert scales (rating scale 1 to 10), single-choice, and multiple-choice questions. The influence of gender, age, position, and institution was also examined.

**Results:**

The personal importance of nutritional medicine among the 225 participants was 8 (7–10) on average, while its importance in the institution where they worked was 5 (3–7). This was rated significantly (p=0.012) higher in university hospitals than in non-university institutions. Obstacles cited include an unclear process for additional training, availability of case seminars and courses, and a lack of support at the management level.

**Conclusion:**

The importance of nutritional medicine at clinical institutions is lower than the personal interest in nutritional medicine and additional training, but this is currently associated with significant obstacles. Structural improvements and greater recognition are needed to keep additional training attractive and to meet the growing demand gap.

## Einleitung

Die Ernährungsmedizin ist ein integraler Bestandteil der Patientenversorgung in allen Fachdisziplinen, jedoch hat sie einen engen thematischen Bezug zur Gastroenterologie. Eine Vielzahl der gastroenterologischen Stoffwechsel- und Tumorerkrankungen geht häufig mit Über- oder Unterernährung einher und muss daher sowohl speziell gastroenterologisch als auch begleitend ernährungsmedizinisch behandelt werden. Bei 20–30% aller stationären Patient*innen zeigt sich eine Mangelernährung, welche durch adäquates Assessment und Ernährungstherapie behandelt werden kann 1 . Das Screening auf Mangelernährung erfordert doch standardisierte Abläufe und Personalkapazitäten. Leider konnte in einer deutschen Studie gezeigt werden, dass in Krankenhäusern ohne Ernährungsteam nur zu 47%, während in Krankenhäusern mit Ernährungsteam zu 83% ein regelmäßiges Screening auf Mangelernährung durchgeführt wird 2 . Die Auswertung der nutritionDay-Daten für Deutschland im 15. DGE-Ernährungsbericht zeigte weiterhin, dass selbst bei schwerer Mangelernährung nur ein kleiner Teil der Patient*innen eine Ernährungsintervention erhalten hat 3 . Dabei ist der positive Effekt einer Ernährungstherapie auf Morbidität, Mortalität und Liegedauer bei mangelernährten Patient*innen hinlänglich belegt 4 5 . Durch den demografischen Wandel ist zudem eine weitere Zunahme der Mangelernährung in Deutschland zu erwarten.

Im Jahr 2023 formierte sich ein Bündnis aus 25 medizinischen Fachgesellschaften und formulierte eine Stellungnahme für eine bessere Struktur- und Prozessqualität bei der Integration der Ernährungsmedizin im Krankenhausalltag. Darin wurde u.a. die Etablierung von Ernährungsteams unter Leitung eines Facharztes mit Zusatzweiterbildung Ernährungsmedizin in Kliniken der Stufe II und Stufe III gefordert 6 . Mit der Anpassung der Musterweiterbildungsordnung 2020 ist die Ernährungsmedizin erstmalig als eigenständige Zusatzweiterbildung verfügbar. Trotz des steigenden Bedarfs sind in Deutschland nur 125 berufstätige Ärzt*innen mit der Zusatzbezeichnung „Ernährungsmedizin“ bei den Kammern registriert 7 . Bereits in der Vergangenheit war eine flächendeckende ernährungsmedizinische Versorgung in deutschen Kliniken nicht zu beobachten und auch heute können wir aufgrund von nur spärlich verfügbaren Daten keine Aussage darüber treffen, wie viele Ernährungsteams in den Kliniken etabliert wurden und wie viele erfolgreiche Absolventen der Zusatzweiterbildung Ernährungsmedizin dem stationären Segment zur Verfügung stehen 8 . Vor dem Hintergrund des allgemeinen Fachkräfte- und Personalmangels unter Ärzt*innen kann mit hoher Wahrscheinlichkeit ebenfalls von einer Minderversorgung durch fehlende Ernährungsmediziner*innen ausgegangen werden 9 .

Wir führten eine prospektive Studie unter jungen Ärzt*innen durch, um den Stellenwert der Ernährungsmedizin sowie das Interesse an der Zusatzweiterbildung Ernährungsmedizin an den verschiedenen medizinischen Einrichtungen zu untersuchen und Hürden zu evaluieren.

## Material und Methodik

### Studienkonzept

Wir haben eine prospektive Umfrage mit dem Online-Fragebogen Tool SoSci Survey Version 3.4.22 (SoSci Survey GmbH München, Deutschland) durchgeführt. Die Einladung zur Umfrage mit einem Link wurde an alle Mitglieder der AG Junge Gastroenterologie, kurz JUGA, per E-Mail am 25.09.2023 verschickt. Am 06.10.2023 erfolgte eine Erinnerung zur Teilnahme. Alle Mitglieder der DGVS unter 41 Jahren oder ohne Facharzt für Gastroenterologie ohne leitende Position sind automatisch JUGA-Mitglieder. Die Beantwortung der Fragen war anonym und freiwillig. Vom 04.09.2023–25.10.2023 wurden 229 Umfragen beantwortet. 4 Fragebögen wurden wegen unvollständiger Beantwortung ausgeschlossen, sodass insgesamt 225 Fragebögen analysiert wurden. Da bei der Umfrage keine personenbezogenen Daten erhoben wurden, bestand keine Beratungspflicht gegenüber der Ethikkommission. Zur gemeinsamen Datennutzung haben wir das Forschungsdatenrepositorium „Zenodo“ (zenodo.org) verwendet. Die Rohdaten stehen ohne Begrenzung unter https://zenodo.org/records/13998690 (DOI: 10.5281/zenodo.13998690) zur Verfügung.

### Fragebogen

Der Fragebogen wurde von den beiden Autor*innen zu gleichen Teilen im Juli 2023 erstellt und im August 2023 getestet. Es handelt sich um einen Fragebogen bestehend aus Single-Choice und Multiple-Choice-Fragen und Fragen mit Bewertungsskala. Hierbei wurde eine Likert-Skala genutzt, wobei die möglichen Antworten als natürliche Zahlen kodiert und aufsteigend angeordnet sind. Es konnten Fragen übersprungen werden. Der Fragebogen gliedert sich in 3 Teile: Fragen 1–4 erfassen demografische Daten (Alter, Geschlecht, Stadium der Weiterbildung, Einrichtung), Fragen 5–6 erfragen den subjektiven einrichtungsbezogenen und persönlichen Stellenwert der Ernährungsmedizin und Fragen 7–10 erheben Daten zur Zusatzweiterbildung Ernährungsmedizin (Weiterbildungsstand, Interesse, Hürden und Unterstützungsmöglichkeiten). Die Abfragelogik verzweigt sich in Abhängigkeit von der Antwort auf den Weiterbildungsstand der Zusatzweiterbildung Ernährungsmedizin. Sollte diese noch nicht abgeschlossen oder begonnen sein, wurde nach Hürden der Zusatzweiterbildung Ernährungsmedizin in Fragen 8–10 gefragt. In Summe beinhaltet der Fragebogen somit 7–10 Fragen, die Beantwortung der Fragen dauert 5–10 Minuten. Der komplette Fragebogen ist als **Supplement 1** zu finden.

### Statistische Analyse

Die statistische Auswertung erfolgte mit SPSS Version 29.0.1.1 (SPSS Inc., Chicago, IL, USA). Die Abbildungen wurden mithilfe von Prism 9.5.0 (GraphPad Software, Inc., La Jolla, USA) erstellt.

Kategorielle Daten sind als Anzahl und Prozent dargestellt, quantitative Ergebnisse als Median und Interquartilsabstand (IQR). Die Normalverteilung der Antworten wurde mittels Kolmogorow-Smirnow-Test geprüft. Je nach Messniveau wurde die statistische Testung mit Hilfe des Mann-Whitney U-Test oder Kruskal-Wallis-Test durchgeführt. Ein p-Wert <0,05 wurde als statistisch signifikant betrachtet.

## Ergebnisse

### Studienkohorte

Von den 225 Teilnehmer*innen waren 125 weiblich (55,6%) und 100 (44,4%) männlich. Der überwiegende Teil der Befragten war 31–40 Jahre (76,4%), gefolgt von 20–30 Jahre (14,2%), 41–50 Jahre (8,9%) und 51–60 Jahre (0,4%) alt. 117 Teilnehmer*innen (52,0%) gaben an, in einer nicht-universitären Klinik tätig zu sein, 76 Teilnehmer*innen (33,8%) an einer Universitätsklinik und 32 Teilnehmer*innen (14,2%) in einer ambulanten Einrichtung. 91 (40,4%) Teilnehmer*innen sind als Oberarzt/ärztin tätig, 68 (30,2%) als Facharzt/ärztin und 66 (29,3%) als Assistenz/ärztin. Eine Zusammenfassung der deskriptiven Daten der Befragten ist in Tab. 1 dargestellt.

**Table:** **Tab. 1** Charakteristika Teilnehmer*innen.

Parameter	Teilnehmer*innen (n=225)
**Geschlecht, w/m**	125 (55,6)/100 (44,6)
**Alter, n**	
20–30 Jahre	32 (14,2)
31–40 Jahre	172 (76,4)
41–50 Jahre	20 (8,9)
51–60 Jahre	1 (0,4)
**Position, n**	
Assistenzarzt/ärztin	66 (29,3)
Facharzt/ärztin	68 (30,2)
Oberarzt/ärztin	91 (40,4)
**Einrichtung, n**	
Ambulante Einrichtung	32 (14,2)
Nicht-universitäre Klinik	117 (52,0)
Universitätsklinikum	76 (33,8)
**Zusatzweiterbildung Ernährungsmedizin**	
absolviert	21 (9,3)
nicht absolviert	35 (15,6)
in Bearbeitung	169 (75,1)

Zum Zeitpunkt der Umfrage gab es 1704 JUGAs. Bei 229 beantworteten Fragebögen ergibt sich eine Rücklaufquote von 13,44%.

### Stellenwert Ernährungsmedizin

**Abb. 1:**
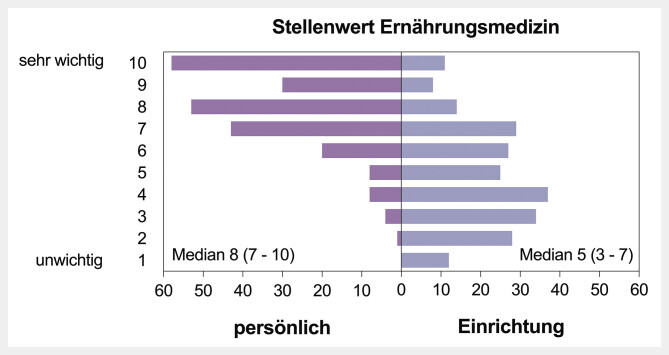
Stellenwert der Ernährungsmedizin – persönlicher Stellenwert (links) vs. Stellenwert in der tätigen Einrichtung (rechts). Dargestellt ist die Anzahl der Befragten.

Der Stellenwert der Ernährungsmedizin wurde auf einer Bewertungsskala von 1 bis 10 beurteilt (unwichtig bis sehr wichtig). Der persönliche Stellenwert der Ernährungsmedizin wurde im Median mit 8 (7–10) angegeben, der Stellenwert der Ernährungsmedizin in der tätigen Einrichtung mit 5 (3–7). Der Vergleich ist grafisch in Abb. 1 dargestellt.

**Abb. 2:**
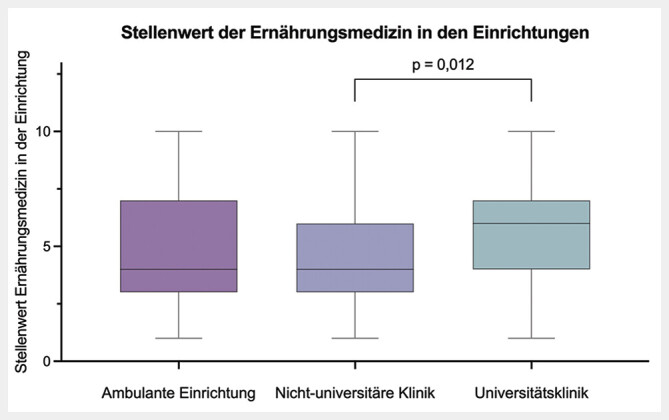
Stellenwert der Ernährungsmedizin in Abhängigkeit von der tätigen Einrichtung.

Es zeigten sich keine signifikanten Unterschiede im persönlichen Stellenwert der Ernährungsmedizin sowie dem Stellenwert der Ernährungsmedizin in der tätigen Einrichtung in Abhängigkeit vom Alter, Geschlecht oder der klinischen Position. Lediglich der Stellenwert der Ernährungsmedizin wurde in Universitätskliniken signifikant höher eingeschätzt als in nicht-universitären Kliniken (6 4 5 6 7 vs. 4 3 4 5 6 , p=0,012) wie in Abb. 2 dargestellt. Unterschiede im persönlichen Stellenwert je nach tätiger Einrichtung zeigten sich jedoch nicht.

### Zusatzweiterbildung Ernährungsmedizin

21 Befragte (9,3%) gaben an, eine Zusatzweiterbildung in der Ernährungsmedizin absolviert zu haben, bei 169 Befragten (75,1%) ist diese noch in Bearbeitung und 35 Befragte (15,6%) haben bisher keine Zusatzweiterbildung in diesem Bereich begonnen.

**Abb. 3:**
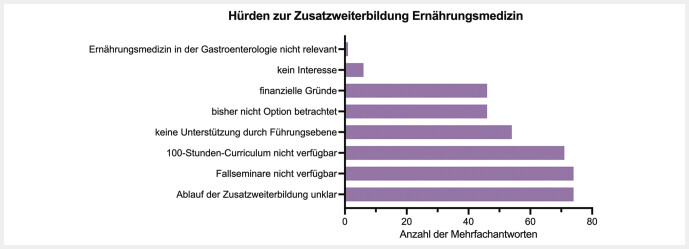
Hürden der Zusatzweiterbildung Ernährungsmedizin. Dargestellt ist die Anzahl der Mehrfachantworten.

In Bezug auf die Hürden zur Zusatzweiterbildung Ernährungsmedizin gaben 74 Teilnehmer*innen an, dass der Ablauf der Zusatzweiterbildung unklar sei und die Fallseminare nicht ausreichend verfügbar sind. 71 Teilnehmer*innen berichteten zudem, dass das 100-Stunden-Curriculum nicht ausreichend verfügbar ist. 54 Teilnehmer*innen berichteten von unzureichender Unterstützung durch die Führungsebene. 46 Teilnehmer*innen gaben an, dass sie die Zusatzweiterbildung Ernährungsmedizin bisher nicht als Option betrachtet haben oder finanzielle Gründe haben. Nur 6 Teilnehmer*innen berichteten von fehlendem Interesse bzw. eine Teilnehmer*in, dass die Ernährungsmedizin in der Gastroenterologie nicht relevant sei. Die Hürden der Zusatzweiterbildung Ernährungsmedizin sind grafisch in Abb. 3 dargestellt.

**Abb. 4:**
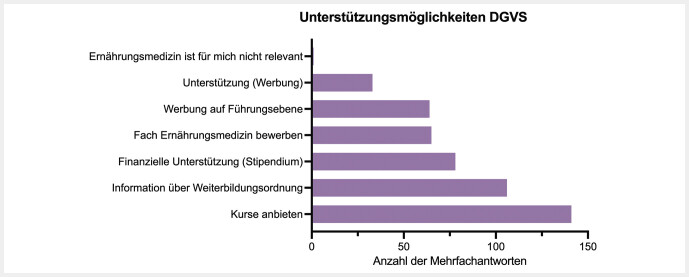
Gewünschte Unterstützungsmöglichkeiten der DGVS von den Teilnehmer*innen zur Absolvierung der Zusatzweiterbildung Ernährungsmedizin. Dargestellt ist die Anzahl der Mehrfachantworten.

Unterstützungsmöglichkeiten der DGVS zur Absolvierung der Zusatzweiterbildung Ernährungsmedizin ( Abb. 4 ) sehen 141 Teilnehmer*innen im Anbieten von Weiterbildungskursen. 106 Teilnehmer*innen wünschen sich Informationen zur Weiterbildungsordnung und 78 Teilnehmer*innen würden sich finanzielle Unterstützung in Form eines Stipendiums von der DGVS wünschen. Werbung auf Führungsebene wurde von 64 Teilnehmer*innen gewünscht, gefolgt von allgemeiner Unterstützung (Werbung) von 33 Teilnehmer*innen. Eine Teilnehmer*in gab an, dass die Ernährungsmedizin für ihn/sie nicht relevant sei.

## Diskussion

Mangelernährung kann durch Unter-, Überernährung oder krankheitsbedingt entstehen und führt zu Gewichtsverlust sowie negativen Auswirkungen auf die Körperzusammensetzung und Funktion 10 . Dadurch wird das klinische Outcome, die Lebensqualität, Liegedauer sowie Funktionalität der Patient*innen signifikant beeinflusst 11 . Durch den demografischen Wandel und damit Zunahme an multimorbiden Patient*innen ist eine weitere Zunahme von Mangelernährung zu erwarten. Sie stellt daher bereits aktuell und auch in Zukunft ein relevantes Gesundheitsproblem dar. Klinische Studien konnten in der Vergangenheit jedoch mehrfach belegen, dass eine individualisierte Ernährungstherapie Mangelernährung entgegenwirken kann und dadurch auch das klinische Outcome der Patient*innen signifikant verbessert 12 13 . Zudem ermöglicht ein systematisches Ernährungsmanagement auch die Senkung der Behandlungskosten, was in Anbetracht der angespannten finanziellen Lage der Krankenkassen wünschenswert wäre. Der gesundheitspolitische Ansatz zur Prävention und damit als Chance die Krankheitslast der Bevölkerung zu senken, findet bereits jetzt in vielen Leitlinien Eingang. Auch Bestrebungen zur Integration der Ernährungsmedizin in die Weiterbildungsordnung Gastroenterologie würden mehr fachkundige Ernährungsmediziner*innen erfordern. Ziel der medizinischen Fachgesellschaften, u.a. DGEM, DGVS, DGIM, ist daher eine flächendeckende Versorgung mit Ernährungsmediziner*innen, um sowohl Präventionsmedizin zu fördern als auch Mangelernährung zu bekämpfen 6 . Aufgrund dieser Entwicklung sollten Hürden zur Absolvierung der Zusatzweiterbildung Ernährungsmedizin dringlich abgebaut werden.

Unsere Umfrage beleuchtet den Stellenwert und die Hürden der Zusatzweiterbildung Ernährungsmedizin unter jungen Ärzt*innen in der Gastroenterologie. Hier wurde zusammenfassend der persönliche Stellenwert der Ernährungsmedizin deutlich höher eingeschätzt als der tatsächliche Stellenwert der Ernährungsmedizin an den unterschiedlichen Einrichtungen, auch wenn dieser an Universitätskliniken signifikant höher beurteilt wurde. Unsere Umfrage zeigt deutliche strukturelle Hürden zur Absolvierung der Zusatzweiterbildung auf.

Die Befragten bewerten den persönlichen Stellenwert der Ernährungsmedizin mit einem hohen Median von 8 auf einer Skala bis 10. Dies unterstreicht die allgemeine Anerkennung der Relevanz der Ernährungsmedizin unter jungen Ärzt*innen. Interessanterweise fällt der wahrgenommene Stellenwert in den jeweiligen Einrichtungen mit einem Median von 5 von 10 deutlich geringer aus. Dies deutet auf eine Diskrepanz zwischen der individuellen Wertschätzung und der institutionellen Prioritätensetzung hin. Wir vermuten, dass die fehlende Erlösrelevanz der Ernährungsmedizin in den Krankenhäusern diese in den Hintergrund drängt. Besonders auffällig ist, dass Universitätskliniken der Ernährungsmedizin einen höheren Stellenwert zuschreiben als nicht-universitäre Kliniken. Dies könnte auf eine höhere Ressourcendichte und ein breiteres Angebot an Weiterbildungsmöglichkeiten in universitären Einrichtungen zurückzuführen sein. Rau et al. konnten in einer prospektiven Befragung deutscher Kliniken bereits zeigen, dass Ernährungsteams signifikant häufiger an größeren Kliniken verfügbar sind und sich anteilig höhere Mitarbeiterzahlen in den Ernährungsteams in größeren Kliniken zeigen 2 . Somit ist insgesamt von einer besseren ernährungsmedizinischen Versorgung an Universitätskliniken auszugehen. Allerdings sollte bei der Interpretation beachtet werden, dass in unserer Umfrage nur 33,8% der Befragten an einer Universitätsklinik tätig sind und dies ggf. zur Verzerrung der Ergebnisse führen kann.

Hinsichtlich des Ablaufs der Zusatzweiterbildung berichteten viele Teilnehmer*innen über unklare Abläufe und eine unzureichende Verfügbarkeit von Fallseminaren und dem 100-Stunden-Curriculum. Hier gilt anzumerken, dass die Inhalte der Zusatzweiterbildung Ernährungsmedizin durch die Bundesärztekammer erst am 28.04.2020 beschlossen wurden. Für die Umsetzung der Weiterbildung inklusive Ausgestaltung, Prüfungszulassung und Übergangsregelung sind die lokalen Landesärztekammern verantwortlich 14 . Erfahrungsgemäß ergeben sich aktuell daraus landespezifische Unterschiede hinsichtlich Prüfungszulassung, Anerkennung von Kursen/Fallseminaren bzw. Möglichkeit der Absolvierung der 6-monatigen Weiterbildungszeit. Auch die Übergangsregelungen können zeitlich und inhaltlich variieren. Diese strukturellen Mängel stellen signifikante Barrieren dar, die die effektive Fortbildung behindern.

Ein weiteres großes Hindernis stellt die fehlende Unterstützung durch die Führungsebene dar. Mehr als ein Viertel der Befragten gab an, dass sie nicht genügend Unterstützung von Vorgesetzten erhielten, was die Teilnahme an der Zusatzweiterbildung erschwere. Diese Faktoren unterstreichen die Notwendigkeit struktureller und administrativer Verbesserungen, um die Teilnahme an der Zusatzweiterbildung zu fördern.

Die Ergebnisse zeigen einen klaren Bedarf an unterstützenden Maßnahmen durch die DGVS und andere relevante Fachgesellschaften. Die meisten Teilnehmer*innen wünschen sich mehr Weiterbildungskurse und detaillierte Informationen zur Weiterbildungsordnung. Finanzielle Unterstützung in Form von Stipendien könnte ebenfalls die Teilnahmebereitschaft erhöhen. Zudem wäre es sinnvoll, auf Führungsebene verstärkt Aufklärungsarbeit für die Bedeutung der Ernährungsmedizin zu leisten, um so die institutionelle Unterstützung zu verbessern.

Unsere Studie hat Limitationen, die wir im Folgenden gerne diskutieren möchten. Eine Einschränkung dieser Studie liegt in der Fokussierung auf eine spezifische Zielgruppe (junge Gastroenterologen*innen unter 41 Jahren oder ohne Facharzt für Gastroenterologie ohne leitende Position der DGVS). Dementsprechend umfasst diese Studie mit 90,6% einen besonders hohen Anteil an jungen Kolleg*innen mit gleichzeitig hohem Anteil an Oberärzt*innen (40,4%). Um die Interessen der älteren Kolleg*innen bzw. Führungspositionen ebenso abzubilden, möchten wir gerne auf die Umfrage zum „Stellenwert der Ernährungsmedizin in der Krankenversorgung, Weiterbildung und Forschung – aus der Führungsperspektive“ verweisen 15 .

Die Rücklaufquote von 13,44% ist vergleichbar mit thematisch ähnlichen Umfragen aus Deutschland 2 . Es ist davon auszugehen, dass an freiwilligen Umfragen dieser Art nur ernährungsmedizinisch interessierte Befragte teilnehmen. Daher ist es nicht verwunderlich, dass 84,4% angaben, aktuell eine Zusatzweiterbildung zum Ernährungsmediziner zu absolvieren oder bereits absolviert haben. So ist hier von einem Selektionsbias auszugehen, da mutmaßlich nur ernährungsmedizinisch interessierte Kolleg*innen an dieser Umfrage teilgenommen haben. Bei der Rücklaufquote ist ebenso zu beachten, dass nicht auszuschließen ist, dass der Link zum Fragebogen auch an ernährungsmedizinisch interessierte Kolleg*innen außerhalb der JUGA weitergereicht wurde und daher eine höhere Rücklaufquote entsteht. Internationale Umfragen zur Ernährungsmedizin zeigen nämlich vergleichsweise schlechtere Rücklaufquoten 16 17 . Das würde ebenso erklären, weshalb es einige Teilnehmer*innen gibt, die <41 Jahre alt sind und bereits einen Facharzt besitzen. Wir gehen jedoch nicht davon aus, dass diese Resultate einen signifikanten Einfluss auf die Gesamtergebnisse haben. Die Verteilung der befragten Ärzt*innen nach der tätigen Einrichtung ist jedoch nahezu repräsentativ für die allgemeine Struktur der Ärzteschaft. Laut Statistik der Bundesärztekammer sind knapp über 50% im stationären Setting tätig, wie ebenso in unserer Umfrage dargestellt 18 . Anzumerken ist außerdem, dass nur gastroenterologische Kolleg*innen unsere Zielgruppe waren und die Motivation und Interessen in anderen Fachgebieten anderweitig verteilt sein könnten. Die Umfrage wurde auch nur unter Mitgliedern einer Fachgesellschaft zirkuliert, zusätzliche Interessengruppen wurden nicht angefragt. Daher ist als schwerwiegende Limitation zusammenfassend anzumerken, dass unsere Ergebnisse möglicherweise nicht repräsentativ für alle medizinischen Fachrichtungen oder Altersgruppen sind.

Weiterhin basiert die Studie auf selbstberichteten Daten, was im Sinne eines Recall Bias zu Verzerrungen führen kann.

## Fazit

Die vorliegende Umfrage verdeutlicht den hohen persönlichen Stellenwert der Ernährungsmedizin unter jungen Gastroenterolog*innen und identifiziert signifikante Hürden bei der Durchführung der Zusatzweiterbildung. Um die Verbreitung und Effektivität der Ernährungsmedizin zu steigern sind strukturelle Verbesserungen, gezielte Unterstützung und eine stärkere institutionelle Anerkennung erforderlich. Langfristig könnte dies zu einer besseren Versorgung der Patient*innen und einer Reduktion der mit Mangelernährung einhergehenden Morbidität und Mortalität führen.

## Zusatzmaterial

Supplementary Material Zusatzmaterial
