# Supplementary material for: Results of a survey on additional qualification in nutritional medicine – need for action recognisable
Source: Z Gastroenterol. 2026 Jan 12;64(3):277–82. [Article in German] doi: 10.1055/a-2737-2113 (PMC13004656; doi:10.1055/a-2737-2113)
Supplement: Supplementary file 1 — Supplementary Material [file 10-1055-a-2737-2113_27372791.pdf]

Supplement 1: Fragebogen
Umfrage JUGA – Ernährungsmedizin

1. Wie alt bist du?
☐ 20 – 30 Jahre
☐ 31 – 40 Jahre
☐ 41 – 50 Jahre
☐ 51 – 60 Jahre

2. Welches Geschlecht hast du?
☐ männlich
☐ weiblich
☐ divers

3. In welchem Stadium der Weiterbildung befindest du dich?
☐ Assistenzarzt/ärztin
☐ Facharzt/ärztin
☐ Oberarzt/ärztin

4. An welcher Einrichtung bis du tätig?
☐ Ambulante Einrichtung
☐ Nicht-universitäre Klinik
☐ Universitäres Klinikum

5. Welchen Stellenwert hat der Bereich Ernährungsmedizin in deiner Klinik?
☐ 1 = findet keine Beachtung im klinischen Alltag
☐ 2
☐ 3
☐ 4
☐ 5
☐ 6
☐ 7
☐ 8
☐ 9
☐ 10 = starke Einbindung im klinischen Alltag

6. Welche Bedeutung hat für dich der Bereich Ernährungsmedizin in der Gastroenterologie?
☐ 1 = unwichtig
☐ 2
☐ 3
☐ 4
☐ 5
☐ 6
☐ 7

Supplement 1: Fragebogen
Umfrage JUGA – Ernährungsmedizin

☐ 8
☐ 9
☐ 10 = sehr wichtig

7. Hast du die Zusatzweiterbildung Ernährungsmedizin absolviert?
☐ ja
☐ nein
☐ in Bearbeitung

→ nur weiter, wenn nein

8. Wie hoch ist dein Interesse daran die Zusatzweitebildung Ernährungsmedizin zu absolvieren?
☐ 1 = kein Interesse
☐ 2
☐ 3
☐ 4
☐ 5
☐ 6
☐ 7
☐ 8
☐ 9
☐ 10 = sehr hoch

9. Welche Hürden halten dich davon ab die Zusatzweiterbildung Ernährungsmedizin zu absolvieren?
☐ kein Interesse
☐ bisher nicht als Option betrachtet
☐ finanzielle Gründe
☐ Fallseminare/100-Stunden-Curriculum nicht verfügbar
☐ Ablauf der Zusatzweiterbildung unklar
☐ keine Unterstützung durch Führungsebene
☐ Ernährungsmedizin ist in der Gastroenterologie nicht relevant
☐ Fallseminare nicht verfügbar

Supplement 1: Fragebogen
Umfrage JUGA – Ernährungsmedizin

10. Wie kann die DGVS dich dabei unterstützen die Zusatzweiterbildung Ernährungsmedizin zu absolvieren?

☐ über Weiterbildungsordnung informieren
☐ Werbung/Infomaterial
☐ Fallseminare/100-Stunden-Curriculum zur Verfügung stellen
☐ Werbung auf Führungsebene
☐ Fach Ernährungsmedizin bewerben
